# New Nanoparticle Formulation for Cyclosporin A: In Vitro Assessment

**DOI:** 10.3390/pharmaceutics13010091

**Published:** 2021-01-12

**Authors:** Amandine Gendron, Natalie Lan Linh Tran, Julie Laloy, Romain Brusini, Aurélie Rachet, Frédéric Gobeaux, Valérie Nicolas, Pierre Chaminade, Sonia Abreu, Didier Desmaële, Mariana Varna

**Affiliations:** 1 Institut Galien Paris-Saclay, Université Paris-Saclay, CNRS UMR 8612, 92296 Châtenay-Malabry, France; amandine.gendron@universite-paris-saclay.fr (A.G.); natalie.tran@universite-paris-saclay.fr (N.L.L.T.); rb.romain.brusini@gmail.com (R.B.); aurelie.rachet@gmail.com (A.R.); didier.desmaele@universite-paris-saclay.fr (D.D.); 2 Namur Nanosafety Centre, Department of Pharmacy, Namur Research Institute for Life Sciences (NARILIS), University of Namur (UNamur), 5000 Namur, Belgium; julie.laloy@unamur.be; 3 Institute for Integrative Biology of the Cell (I2BC), Université Paris-Saclay, CEA, CNRS, 91198 Gif-sur-Yvette, France; 4 CEA, CNRS, NIMBE, Université Paris-Saclay, CEA-Saclay, 91191 Gif sur Yvette, France; Frederic.GOBEAUX@cea.fr; 5 Ingénierie et Plateformes au Service de l’Innovation (IPSIT), UMS IPSIT Université Paris-Saclay—US 31 INSERM—UMS 3679 CNRS, Plate-forme d’imagerie cellulaire MIPSIT, 92290 Châtenay-Malabry, France; valerie.nicolas@universite-paris-saclay.fr; 6 Lipides: Systèmes Analytiques et Biologiques, Université Paris-Saclay, 92296 Châtenay-Malabry, France; pierre.chaminade@universite-paris-saclay.fr (P.C.); sonia.abreu@universite-paris-saclay.fr (S.A.)

**Keywords:** squalene, cyclosporin A, bioconjugate, cardiac cell line, cytotoxicity, cellular uptake

## Abstract

Cyclosporin A (CsA) is a molecule with well-known immunosuppressive properties. As it also acts on the opening of mitochondrial permeability transition pore (mPTP), CsA has been evaluated for ischemic heart diseases (IHD). However, its distribution throughout the body and its physicochemical characteristics strongly limit the use of CsA for intravenous administration. In this context, nanoparticles (NPs) have emerged as an opportunity to circumvent the above-mentioned limitations. We have developed in our laboratory an innovative nanoformulation based on the covalent bond between squalene (Sq) and cyclosporin A to avoid burst release phenomena and increase drug loading. After a thorough characterization of the bioconjugate, we proceeded with a nanoprecipitation in aqueous medium in order to obtain SqCsA NPs of well-defined size. The SqCsA NPs were further characterized using dynamic light scattering (DLS), cryogenic transmission electron microscopy (cryoTEM), and high-performance liquid chromatography (HPLC), and their cytotoxicity was evaluated. As the goal is to employ them for IHD, we evaluated the cardioprotective capacity on two cardiac cell lines. A strong cardioprotective effect was observed on cardiomyoblasts subjected to experimental hypoxia/reoxygenation. Further research is needed in order to understand the mechanisms of action of SqCsA NPs in cells. This new formulation of CsA could pave the way for possible medical application.

## 1. Introduction

Cyclosporin A (CsA) is a well-known immunosuppressive drug identified and isolated from *Tolypocladium inflatum* or *Cylindrocapron lucidum* [1]. It is a hydrophobic neutral cyclic peptide with the molecular formula C_62_H_111_N_11_O_12_, and a molecular weight of 1202.6 Da [2,3,4]. This peptide is composed of eleven amino acid residues, seven of which are N-methylated. According to the Biopharmaceutical Classification Systems, CsA is classified as a class II compound that is known to have extremely low aqueous solubility (6.6 µg/mL) and high lipophilicity (Log P = 3) [5]. As a result of its immunosuppressive capabilities, this peptide was initially approved to prevent graft rejection in organ transplantation [6,7], but other clinical applications are now employed, such as in psoriasis or in atopic dermatitis treatment. In addition, clinical trials for the treatment of other types of diseases such as rheumatoid arthritis, ulcerative colitis, or ophthalmic applications are currently under investigation [8,9,10,11,12]. CsA was initially marketed as an oil-based formulation for oral administration (Sandimmune Neoral^®^ from Novartis); however, nowadays, other commercial formulations of CsA are available on the market. Restasis^®^ (from Allergan) marketed in 2003 in the USA, containing 0.5 mg/mL of CsA, is an anionic emulsion of castor oil in water. Ikervis^®^ (from Santen), marketed in 2015 in Europe, is a nanoemulsion containing 1 mg/mL of CsA [5,13].

CsA possesses a selective action on T lymphocytes while avoiding myelotoxicity [14]. The mechanisms of action of this peptide are relatively well known. CsA binds to cyclophilin A, with high affinity forming a complex that binds to calcineurin and inhibits it. The formation of this complex is necessary for its immunosuppressive effect. Indeed, it prevents the translocation of the nuclear factor of activated T cells (NF-AT) from the cytoplasm to the nucleus, thereby blocking the early expression of interleukins (IL), in particular IL-2, IL-3, IL-4, and tumor necrosis factor α (TNFα) [2,15]. More recently, CsA has shown its potential in IHD treatment. IHD often result from the negative effects of myocardial ischemia/reperfusion injuries. The opening of the mitochondrial permeability transition pore (mPTP) is one of the critical mechanisms leading to injuries after reperfusion and ultimately to cardiomyocyte death. Numerous preclinical studies have shown that CsA can inhibit mPTP opening by binding to cyclophilin D, which is a major component of mPTP [16,17,18].

In 2008, a proof-of-concept clinical study conducted by Piot et al. [19] first demonstrated that a single intravenous bolus of CsA 2.5 mg/kg administered 10 min prior to reperfusion could limit the size of myocardial infarct in ST-elevation myocardial infarction (STEMI) patients. However, these results were subsequently mitigated when conducted in larger randomized, double blind, multicenter trials [20,21]. These contradictory results could be explained by the fact that when injected into the blood, CsA is distributed throughout the body and accumulates in lower amounts in the heart due to its low cell permeability.

To circumvent the above-mentioned limitations, a nanoparticle (NP) formulation could be a promising approach to increase the bioavailability, solubility, and circulation time as well as deliver this peptide into targeted tissues and organs. To this end, several CsA-loaded nanoparticles have been developed, including lipid-based nanoparticles [22,23], microspheres [24,25], or polymeric nanoparticles [26,27]. However, these formulations are mainly developed for oral or local administration (i.e., ocular, cutaneous) and possess some limitations such as low entrapment efficacy, inconsistent drug release, and sometimes toxicity [28,29,30].

To improve on these issues, we propose a new nanomedicine based on the use of squalene (Sq), a natural, biocompatible, and biodegradable lipid [31]. The conjugation of therapeutic molecules to squalene has been shown to enhance blood circulation time [32], provide interesting targeting properties [33,34,35], and lower toxicity [36]. More recently, we reported the development of multidrug nanoparticles made of squalene conjugated to adenosine and encapsulating α-tocopherol as antioxidant for the mitigation of uncontrolled inflammation [37]. Furthermore, we have demonstrated the scaling-up synthesis and formulation of Sq-based NPs from laboratory to industrial scale while maintaining control of their characteristics [38].

In the present study, we have engineered a new type of nanomedicine obtained after the covalent conjugation of squalene to cyclosporin A. The bioconjugate as well as the nanoparticles obtained after nanoprecipitation in an aqueous medium were thoroughly characterized. Finally, the cytotoxicity, the cellular uptake, and the cardioprotective effect were assessed in vitro on two cardiac cell lines.

## 2. Materials and Methods

### 2.1. Materials

Chemicals obtained from commercial suppliers were used without further purification. Cyclosporin A (CsA) was supplied by Carbosynth (Berkshire, UK). MilliQ water (resistivity of 18.2 MΩ·cm) was obtained in house and purified with a Millipore system. CDCl_3_ and C_6_D_6_ were purchased from Eurisotop (Saint-Aubain, France). The MCEC cell line was purchased from Tebu-Bio (Le Perray en Yvelines, France). H9c2 cell line, Dulbecco’s Modified Eagle’s Medium (DMEM) D6429 and D5796 high glucose, D5030 (without glucose), Dulbecco’s Phosphate-Buffered Saline (PBS), 4-(2-hydroxyethyl)-1-piperazineethanesulfonic acid (HEPES; 1 M, pH 7.0–7.6), trypsin-ethylenediamine tetraacetic acid (EDTA), Triton™ X-100, penicillin–streptomycin, phalloidin–Atto 488, D-(+)-glucose, thiazolyl blue tetrazolium bromide (MTT), methanol, ethyl acetate, squalene, sodium chloride, chloroacetic anhydride, cesium hydrogencarbonate and Cameo syringe filters (1 µm) were purchased from Sigma-Aldrich (Lyon, France). Lactate dehydrogenase (LDH) tests were purchased from Promega (Paris, France). Absolute ethanol, HPLC grade water, dimethyl sulfoxide (DMSO) and diethyl ether were purchased from VWR chemicals (Paris, France). Fetal Bovine Serum (FBS) was obtained from Life Technologies (Illkirch-Graffenstaden, France). Cholesteryl 4,4-difluoro-5-(4-methoxyphenyl)-4-bora-3a,4a-diaza-s-Indacene-3-undecanoate (CholEsteryl BODIPY™, 542/563 C11) was purchased from Thermo Fisher Scientific (Illkirch Graffenstaden, France). Ibidi µ-Slide 8-well plates and immunofluorescence mounting medium containing 4′,6-diamidino-2-phénylindole (DAPI) were obtained from CliniSciences (Nanterre, France). Paraformaldehyde (PFA) was purchased from Carl Roth (Lagny sur Marne, France).

### 2.2. Synthesis of the SqCsA Conjugate

#### 2.2.1. General

Infrared (IR) spectra were obtained as solid or neat liquid on a Fourier Transform Bruker Vector 22 spectrometer. Only significant absorptions are listed. Optical rotations were measured on a Perkin-Elmer 241 Polarimeter at 589 nm. The ^1^H and ^13^C nuclear magnetic resonance (NMR) spectra were recorded on Bruker Avance 300 (300 MHz and 75 MHz for ^1^H and ^13^C, respectively) or Bruker Avance 400 (400 MHz and 100 MHz for ^1^H and ^13^C, respectively) spectrometers. Recognition of methyl, methylene, methine, and quaternary carbon nuclei in ^13^C NMR spectra rests on the *J*-modulated spin-echo sequence. Mass spectra were recorded on a Bruker Esquire-LC. High-resolution mass spectra were recorded on a electrospray ionization time-of-flight (ESI/TOF) (LCT, Waters, Milford, MA, USA) LC-spectrometer. Analytical thin-layer chromatography (TLC) was performed on Merck silica gel 60F_254_ glass precoated plates (0.25 mm layer). Column chromatography was performed on Merck silica gel 60 (230–400 mesh ASTM). Toluene, pyridine, dimethylformamide (DMF), and CH_2_Cl_2_ were distilled from calcium hydride, under a nitrogen atmosphere. All reactions involving air- or water-sensitive compounds were routinely conducted in glassware, which was flame-dried under a positive pressure of nitrogen.

#### 2.2.2. Synthesis of Cyclosporin A Chloroacetic Ester (CsA-COCH_2_Cl)

Chloroacetic anhydride (600 mg, 3.5 mmol) was added portion-wise to a solution of cyclosporin A (500 mg, 0.42 mmol) in pyridine (1.25 mL, 16 mmol). The thick reaction mixture was stirred at 50 °C for 3 h under nitrogen flow and monitored by TLC with ethyl acetate/cyclohexane (80:20) as eluent. The reaction mixture was taken into water (7 mL) and extracted with Et_2_O (3 × 20 mL). The combined organic layers were washed with brine (1 × 20 mL), dried over MgSO_4_, and concentrated under reduced pressure. The crude product was purified by silica gel chromatography eluted with ethyl acetate/cyclohexane (80:20) to obtain the cyclosporin A chloroacetic ester (290 mg, 55% yield) as a white solid. ^1^H NMR (C_6_D_6_, 300 MHz) *δ*: 8.69 (d, *J* = 9.6 Hz, 1H, N*H*-2), 8.24 (d, *J* = 6.9 Hz, 1H, N*H*-7), 7.90 (d, *J* = 7.8 Hz, 1H, N*H*-8), 7.48 (d, *J* = 9.3 Hz, 1H, N*H*-5), 6.05 (d, *J* = 11.1 Hz, 1H, *H*-1β), 5.95–5.85), (m, 1H-9α), 5.84 (d, *J* = 11.1 Hz, 1H, *H*-1α), 5.65–5.30 (m, 5H), 5.01 (t, *J* = 9.3 Hz, 1H), 5.05–4.95 (m, 2H), 4.85–4.72 (m, 2H), 4.56 (quint, *J* = 6.9 Hz, 1H, H-7α), 4.39 (d, *J* = 14.7 Hz, 1H, C*H*_2_Cl), 4.02 (d, *J* = 13.5 Hz, 1H, *H*-3α), 3.95 (d, *J* = 14.7 Hz, 1H, C*H*_2_Cl), 3.33 (s, 3H, NC*H_3_*), 3.29 (s, 3H, NC*H_3_*), 3.16 (s, 3H, NC*H_3_*), 3.09 (s, 3H, NC*H_3_*), 2.95 (s, 3H, NC*H_3_*), 2.78 (s, 3H, NC*H_3_*), 2.62 (s, 3H, NC*H_3_*), 2.55–2.15 (m, 10H), 1.80 (d, *J* = 4.5 Hz, 3H, C*H_3_*CH=), 1.51 (d, *J* = 7.2 Hz, 3H, C*H*_3_CH-8), 1.95–1.30 (m, 4H), 1.30–1.18 (m, 13H), 1.13 (d, *J* = 6.3 Hz, 3H), 1.02 (d, *J* = 7.2 Hz, 3H), 0.99 (d, *J* = 6.6 Hz, 6H), 0.91 (d, *J* = 6.3 Hz, 6H), 0.90–0.80 (m, 11 H), 0.74 (d, *J* = 6.3 Hz, 3H), 0.59 (d, *J* = 6.3 Hz, 3H) ppm; ^13^C NMR (75 MHz, CDCl_3_) *δ*: 173.97 (C, CO), 173.55 (C, CO), 173.06 (C, CO), 172.83 (C, CO), 171.51 (C, CO), 171.33 (C, CO), 171.08 (C, CO), 170.92 (C, CO), 169.97 (C, CO), 167.82 (C, CO), 167.31 (C, CO), 128.93 (CH, CH_3_H*C*=CH), 126.74 (CH, CH_3_HC=*C*H), 75.50 (CH, *C*-1β), 58.89 (CH), 57.50 (CH), 55.87 (CH), 55.32 (CH), 54.84 (CH), 54.36 (CH), 50.15 (CH_2_, CH_2_Cl), 48.88 (CH), 48.33 (CH), 48.04 (CH), 44.76 (CH), 40.97 (CH_2_), 40.88 (2CH_2_), 39.38 (CH_3_, N*CH_3_*), 39.31 (CH_2_), 37.08 (CH_2_), 35.93 (CH_2_), 33.77 (CH_2_), 33.23 (CH_3,_ N*C*H_3_), 32.32 (CH), 31.70 (CH), 31.43 (CH_3,_ N*CH_3_*), 31.39 (CH_3_, N*CH_3_*), 30.35 (CH), 30.01 (CH), 29.75 (CH_3_), 29.46 (CH_3_), 25.09 (CH_2,_ *C*-2β), 24.87 (CH), 24.73 (CH_3_), 24.64 (CH_3_), 24.30 (CH_3_), 23.93 (CH_3_), 23.86 (CH_3_), 23.71 (CH_3_), 23.62 (CH_3_), 21.89 (CH_3_), 21.35 (CH_3_), 21.24 (CH_3_), 20.73 (CH_3_), 19.73 (CH_3_), 18.69 (CH_3_), 18.25 (CH_3_), 17.84 (CH_3_), 17.79 (CH_3_), 17.68 (CH_3_), 15.12 (CH_3,_ C-7γ), 9.97 (CH_3,_ C-2γ) ppm; MS (ESI+) *m/z* (%): 1300.8 (100) [M+Na]^+^, 1278.8 (5) [M+H]^+^, 666.9 (70) [M+Na+K]^2+^, 661.9 (85) [M+2Na]^2+^, 650.9 (3) [M+2H]^2+^.

#### 2.2.3. Synthesis of 1,1′,2-Trisnorsqualenic Acid Cesium Salt

Cesium hydrogencarbonate (296 mg, 1.52 mmol) was added to a solution of trisnorsqualenic acid (580 mg, 1.45 mmol) in methanol (20 mL) [39,40]. The reaction mixture was stirred at room temperature for 2 h and concentrated under reduced pressure. The waxy solid obtained was used directly in the next step.

#### 2.2.4. Synthesis of SqCsA Bioconjugate (SqCsA)

First, 1,1′,2-trisnorsqualenic acid cesium salt (122 mg, 0.23 mmol) was added to a solution of cyclosporin chloroacetic ester (117 mg, 0.092 mmol) in DMF (250 μL). The reaction was stirred at 50 °C for 24 h. Once the reaction completed, the product was concentrated under reduced pressure and taken into 5 mL of water and extracted with ethyl acetate (3 × 10 mL). The combined organic layers were washed with brine (1 × 5 mL), dried over MgSO_4_, and concentrated under reduced pressure. The crude product was purified by silica gel chromatography eluted with ethyl acetate/cyclohexane (50:50) to obtain the SqCsA bioconjugate (94 mg, 62% yield) as a colorless foam. [α]_D_ -212.8 (EtOH, c = 0.5); IR (neat, cm^−1^) *υ*: 3400–3200, 2963, 2933, 2873, 1769, 1750, 1677, 1620, 1539, 1517, 1472, 1414, 1388, 1383, 1367, 1319, 1290, 1267, 1211, 1193, 1149, 1097, 984, 968; ^1^H NMR (C_6_D_6_, 400 MHz) only the major conformer is described *δ*: 8.73 (d, *J* = 9.6 Hz, 1H, N*H*-2), 8.30 (d, *J* = 7.2 Hz, 1H, N*H*-7), 7.93 (d, *J* = 7.6 Hz, 1H, N*H*-8), 7.48 (d, *J* = 9.6 Hz, 1H, N*H*-5), 6.11 (d, *J* = 11.2 Hz, 1H, *H*-1β), 5.95–5.85 (m, 2H, H-1α, H-9α), 5.61 (dt, *J* = 12.0 Hz, *J* = 3.6 Hz, 1H, H-4α), 5.34–5.16 (m, 5H, *H*C=C(Me)), 5.10 (d, *J* = 15.6 Hz, 1H, SqCO_2_C*H*_2_CO), 5.02 (t, *J* = 9.6 Hz, 1H), 4.86 (d, *J* = 11.2 Hz, 1H, H-5α), 4.82 (d, *J* = 15.6 Hz, 1H, SqCO_2_C*H*_2_CO), 4.77 (quint, *J* = 7.2 Hz, 1H, H-8α), 4.57 (quint, *J* = 7.2 Hz, 1H, H-7α), 4.03 (d, *J* = 13.6 Hz, 1H, *H*-3α), 3.46 (s, 3H, NC*H_3_*), 3.28 (s, 3H, NC*H_3_*), 3.16 (s, 3H, NC*H_3_*), 3.10 (s, 3H, NC*H_3_*), 2.98 (s, 3H, NC*H_3_*), 2.82 (s, 3H, NC*H_3_*), 2.63 (s, 4H, H-5β, NC*H_3_*), 2.50–1.90 (m, 32H), 1.90–1.70 (m, 6H), 1.68 (s, 3H, =C(C*H*_3_)_2_), 1.63 (s, 3H, =C(C*H*_3_)CH_2_), 1.61 (s, 3H, =C(C*H*_3_)CH_2_), 1.59 (s, 3H, =C(C*H*_3_)CH_2_), 1.57 (s, 3H, =C(C*H*_3_)CH_2_), 1.54 (d, *J* = 7.2 Hz, 3H, H-7β), 1.53–1.25 (m, 3H), 1.45 (s, 3H, =C(C*H*_3_)CH_2_), 1.35 (d, *J* = 6.8 Hz, 3H), 1.25 (t, *J* = 7.0 Hz, 9H), 1.21 (d, *J* = 6.4 Hz, 3H), 1.14 (d, *J* = 6.8 Hz, 3H), 1.07 (d, *J* = 6.4 Hz, 3H), 1.04 (d, *J* = 6.8 Hz, 3H), 0.99 (d, *J* = 6.4 Hz, 3H), 0.94–0.90 (m, 6H), 0.88–0.82 (m, 9H), 0.76 (d, *J* = 6.8 Hz, 3H, H-11γ), 0.60 (d, *J* = 6.4 Hz, 3H, H-11γ) ppm; ^13^C NMR (C_6_D_6_, 100 MHz) *δ*: 174.64 (C, CO), 173.98 (C, CO), 173.58 (C, CO), 173.06 (C, CO), 172.42 (C, Sq*C*O_2_CH_2_CO), 171.69 (C, CO), 171.49 (C, CO), 171.21 (C, CO), 171.01, 170.87 (C, CO), 169.79 (C, CO), 168.24 (C, CO-2), 168.20 (C, SqCO_2_CH_2_*C*O), 135.17 (2C, CH_2_(CH_3_)*C*=), 134.97 (C, CH_2_(CH_3_)*C*=), 133.46 (C, CH_2_(CH_3_)*C*=), 131.10 (C, (CH_3_)_2_*C*=), 130.07 (CH, CH_3_HC=*C*H), 126.78 (CH, CH_3_H*C*=CH), 125.44 (CH, CH_2_(CH_3_)C=*C*H), 125.00 (CH, CH_2_(CH_3_)C=*C*H), 124.92 (CH, CH_2_(CH_3_)C=*C*H), 124.87 (CH, CH_2_(CH_3_)C=*C*H), 124.84 (CH, CH_2_(CH_3_)C=*C*H), 75.01 (CH, *C*-1β), 61.30 (CH_2_, SqCO_2_*C*H_2_CO_2_), 59.06 (CH), 57.80 (CH), 56.42 (CH), 55.48 (CH), 55.02 (CH), 54.73 (CH), 49.51 (CH_2_, *C*-3α), 49.05 (CH), 48.51 (CH), 48.23 (CH), 45.00 (CH), 41.32 (CH_2_), 40.23 (2CH_2_), 40.03 (CH_2_), 39.92 (CH_2_), 39.08 (CH_3_, N*C*H_3_), 37.71 (CH_2_), 36.29 (CH_2_), 34.76 (CH_2_), 34.67 (CH_2_), 33.68 (CH), 33.06 (CH_2_), 32.59 (CH_3_, N*C*H_3_), 32.09 (CH), 31.54 (CH_3_, N*C*H_3_), 30.98 (CH_3_, N*C*H_3_), 30.82 (CH_3_, N*C*H_3_), 30.15 (CH_2_), 30.11 (CH_3_, N*C*H_3_), 29.79 (CH), 29.62 (CH_3_, N*C*H_3_), 28.76, (2CH_2_), 27.27 (CH_2_), 27.14 (2CH_2_), 25.87 (CH_3_, =C(*C*H_3_)_2_), 25.53 (CH_2_), 25.19 (CH_3_), 25.07(CH_3_), 25.03 (CH_3_), 24.99 (CH_3_), 24.87 (CH_3_), 24.14 (CH_3_), 24.04 (CH_3_), 23.94 (CH_3_), 23.76 (CH_3_), 22.06 (CH_3_), 21.69 (CH_3_), 21.39 (CH_3_), 20.25 (CH_3_), 19.95 (CH_3_), 18.59 (CH_3_), 18.36 (CH_3_), 17.94 (CH_3_), 17.77 (CH_3_), 17.55 (CH_3_), 16.20 (CH_3_), 16.15 (CH_3_), 15.90 (CH_3_), 15.31 (CH_3_), 10.17 (CH_3,_ C-2γ) ppm; MS (ESI+) *m/z* (%): 1665.2 (92) [M+Na]^+^, 1643.2 (48) [M+H]^+^, 844.1 (45) [M+2Na]^2+^, 833.1 (25) [M+H+Na]^2+^, 822.1 (17) [M+2H]^2+^. HRMS (–ESI): calcd. for C_91_H_155_O_15_N_11_Na: 1665.1596; found 1665.1569.

### 2.3. Preparation and Characterization of SqCsA Nanoparticles

SqCsA NPs were prepared by nanoprecipitation, which was a technique previously validated in our laboratory with other drugs such as adenosine [31,32,37]. Briefly, SqCsA was dissolved in absolute ethanol (6 mg/mL) and added dropwise under vigorous stirring to 1 mL of a 5% (*w*/*v*) dextrose solution to give an aqueous suspension of SqCsA NPs at 2 mg/mL. The ethanol was completely evaporated using a Rotavapor (80 rpm, 40 °C, 43 mbar), and the suspension was filtered through a 1 µm syringe filter. Similarly, the NPs aqueous suspension at 4 mg/mL was obtained by adding the ethanolic suspension to 2 mL of a 2.5% (*w*/*v*) dextrose solution. Evaporation of ethanol and 1 mL of MilliQ water resulted in a 5% dextrose solution with NPs concentrated at 4 mg/mL. Fluorescent NPs were prepared using the same protocol, although 0.1% (*w*/*w*) of CholEsteryl BODIPY™ C11 was added to the ethanolic phase. NPs size (hydrodynamic diameter), polydispersity index, and surface charge (zeta potential) were measured using a Zetasizer Nano ZS (173° scattering angle, 25 °C Malvern). For size and zeta potential measurements, 50 μL of NPs were respectively mixed with 950 µL of MilliQ water and NaCl 1 mM before filling the measurement cells. The mean zeta potential for each preparation resulted from the mean of three measurements in automatic mode, followed by the application of the Smoluchowski equation.

### 2.4. Morphology by CryoTEM

The morphology of SqCsA NPs was observed by cryogenic transmission electron microscopy (cryoTEM) as previously published [38]. Briefly, a few drops of the NPs suspension (2 mg/mL) were deposited on EM grids covered with a holey carbon film (Quantifoil R2/2) previously treated with a plasma glow discharge. The observations were conducted at low temperature (−180 °C) on a JEOL 2010 FEG microscope operated at 200 kV, and images were recorded with a Gatan camera.

### 2.5. Stability of NPs

The stability of the NPs suspensions was assessed by evaluating the size and surface charge measurement by Zetasizer Nano ZS at different times: day 0, 1, 2, 5, 7, 9, 12, 14, 28 and different storage conditions: 4 °C and room temperature (RT). At each time point, 3 measurements were taken.

### 2.6. HPLC Analysis

#### 2.6.1. Sample Preparation

Two hundred microliters of SqCsA NPs (2 mg/mL) were incubated in 427 µL of nonfiltered FBS in 21 hemolysis tubes for 0, 0.5, 1, 4, 18, 24, and 48 h (3 for each time-point). Four milliliters of a mixture of diethyl ether and methanol (95/5 *v*/*v*) were added to each hemolysis tube. After vortex mixing for 30 s, the tubes were stirred for 1 h in a water bath at 37 °C. Samples were vortex-mixed for 30 s and centrifuged for 30 min at 1000 G. The organic phase was recovered, and the aqueous phase was re-extracted using to the same protocol. Then, the pooled organic phases of each tube were evaporated to dryness under nitrogen flow at room temperature. Ultimately, it was resuspended in 400 µL of methanol, vortexed for 30 s, and 10 µL were injected into the HPLC.

Calibration curves were obtained using 11 concentrations between 0.001 and 0.1 mg/mL for CsA and squalenic acid and between 0.01 and 1 mg/mL for SqCsA bioconjugate. We verified that all points of the calibration curves were greater than or equal to 10 times the background noise or limit of quantification (LOQ).

#### 2.6.2. Chromatographic System

HPLC analysis was carried out on a Dionex Ultimate 3000 (Thermo Fisher Scientific) apparatus, equipped with a Corona CAD^®^ system (ESA, Chelmsford, MA, USA), and the signal was acquired with a Chromeleon data station (Thermo Fisher Scientific). Corona CAD^®^ settings were as follows: range 500 pA, no filter, and air pressure 35 psi. Chromatographic separation was performed on a Kromasil C8 column (150 × 4.6 mm, 5 µm particle size) at room temperature. The mobile phase system consisted of a mixture (90/10 *v*/*v*) of methanol and HPLC grade water (A) and ethyl acetate (B), and the flow rate was 1 mL/min. A gradient elution was used: from 100% of A to 100% of B over a period of 15 min and maintained at 100% of B during 7 min. Then, the column was equilibrated with 100% A for 10 min.

### 2.7. Cell Culture

Immortalized Mouse Cardiac Endothelial Cells (MCEC) and rat cardiomyoblasts (H9c2) cells were cultured in 75 cm^2^ culture flasks containing 12 mL of a complete medium at 37 °C in a 5% CO_2_ humidified incubator. The complete medium was composed of DMEM D6429 with 10% FBS and 1% penicillin–streptomycin for H9c2 cells and DMEM D5796 with 5% FBS, 1% penicillin–streptomycin and 1% HEPES for MCEC.

### 2.8. Cytotoxicity of SqCsA NPs

The cytotoxicity of SqCsA NPs was evaluated on MCEC and H9c2 cells using an MTT assay. Cells were seeded in 96-well plates at 6000 cells per well (MCEC) and 10,000 cells per well (H9c2) for 24 h. Subsequently, cells were incubated at 37 °C in a 5% CO_2_ humidified incubator with concentrations of SqCsA NPs ranging from 0.6 to 60 µg/mL and 44 µg/mL of free CsA (equivalent maximal dose, dissolved in ethanol) for 4 and 24 h. After incubation, an MTT solution (5 mg/mL in PBS) was added to each well for 2 h. Then, the culture medium was removed, and 200 µL of DMSO were used in each well to dissolve formazan crystals. Plates were stirred for 5 min at 40 rpm on a plate shaker, and the absorbance was measured at 570 nm using an ELISA plate reader. Cell viability was expressed as a percentage of the control wells (untreated cells).

### 2.9. Cell Uptake of Fluorescently Labeled SqCsA NPs

MCEC and H9c2 cells were seeded in 8-well Ibidi plates at a density of 15,000 cells per well. Cells were left to adhere for 24 h and then incubated with 12 µg/mL or 60 µg/mL of fluorescent SqCsA NPs for 2, 18, and 24 h in a 5% CO_2_ humidified incubator at 37 °C. After incubation, wells were washed with PBS, and then, cells were fixed with 4% PFA for 5 min and permeabilized with 0.1% Triton™ X-100 for 3 min. The cytoskeleton of actin was stained with phalloidin for 1 h at room temperature, and nuclei were stained with DAPI contained in the mounting medium 15 min before observation. Images were obtained using an inverted Confocal Laser Scanning Microscope (CLSM) Leica TCS SP8 with an HC PL APO CS2 63×/1.40 oil immersion objective lens. The instrument was equipped with a 405 nm diode for DAPI (nuclei) excitation and a WLL Laser (488 nm excitation for phalloidin-Atto 488 and 542 nm for CholEsteryl BODIPY™ NPs). Blue, green, and red fluorescence emissions were collected respectively with 410–460, 505–550, and 560–760 nm wide emission slits using a sequential mode. The pinhole was set at 1.0 Airy unit giving an optical slice thickness of 0.89 µm. Twelve-bit numerical images were acquired using Leica SP8 LAS X software (Version 3.6; Leica, Wetzlar, Germany)

### 2.10. Cardioprotective Effect Assessment

To assess whether the nanoparticles have cardioprotective effects, MCEC and H9c2 cells were seeded on 96-well plates as described above. Twenty-four hours after plating, the cells were treated for another 24 h with SqCsA NPs. Controls were performed simultaneously: free CsA, Sq NPs, and untreated cells. The following day, the cells were washed with PBS and incubated with glucose-free medium and FBS under hypoxic conditions (1% O_2_) in a dedicated incubator (Panasonic, France). Three conditions were tested: 6 h of hypoxia (6 h), 6 h of hypoxia and 30 min of reoxygenation (6 h + 30 min), and 6 h of hypoxia and 1 h of reoxygenation (6 h + 1 h). Reoxygenation was obtained by adding enriched media specific for each cell type followed by incubation under normoxia (17% O_2_ and 5% CO_2_). We assessed the protective effect by using MTT and LDH tests.

### 2.11. Statistical Analysis

Data were expressed as mean ± standard deviation (SD). Statistics were computed using the GraphPad Prism 7.0 software (GraphPad Software, Inc., San Diego, CA, USA). Statistical differences between two groups were evaluated using the unpaired Student *t* test. A value of *p* < 0.05 was considered significant.

## 3. Results and Discussion

### 3.1. Synthesis of the SqCsA Conjugate

**Figure 1:**
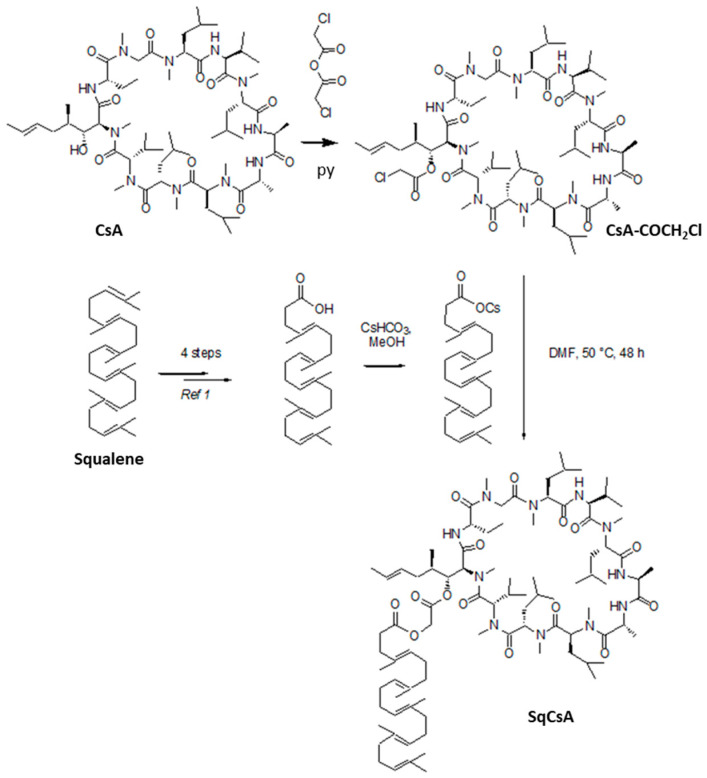
Synthetic scheme of the squalene cyclosporin A (SqCsA) conjugate. The squalene chain was introduced on the side chain of the MeBmt residue through a glycolate linker by chloroacetylation of the CsA followed by S_N_2 displacement of the chloride using squalenic acid cesium salt.

Many CsA conjugates have been previously tailored to increase its water solubility or cellular uptake. Other than a few derivatives made by functionalization of the double bond of the 2-butenyl-4-N-dimethyl-L-threonine residue (MeBmt) [41,42], most bioconjugates were prepared by using the free hydroxyl group on the side chain of the MeBmt amino acid. However, this group embedded in the CsA core turned out to be highly hindered and poorly reactive. For instance, the conversion of CsA into its corresponding chloroformate derivative using phosgene required a one-week reaction with a large excess of reagent [43]. Extensive research in the field led to the discovery that condensation with chloro- or bromoacetic anhydride constituted the most efficient way for the derivatization of CsA [44,45,46]. In the present case, the chloroacetate derivative was chosen as the starting material to obtain the squalene conjugate. Thus, CsA was first condensed with chloroacetic anhydride to give the known chloroacetate [44,45,46], which further reacted with the cesium salt of 1,1′,2-trisnorsqualenic acid [39,40] through S_N_2 type reaction (Figure 1). Accordingly, the SqCsA conjugate was obtained with an overall yield of 35%. The compound appeared as a colorless amorphous solid that is soluble only in organic solvents.

**Figure 2:**
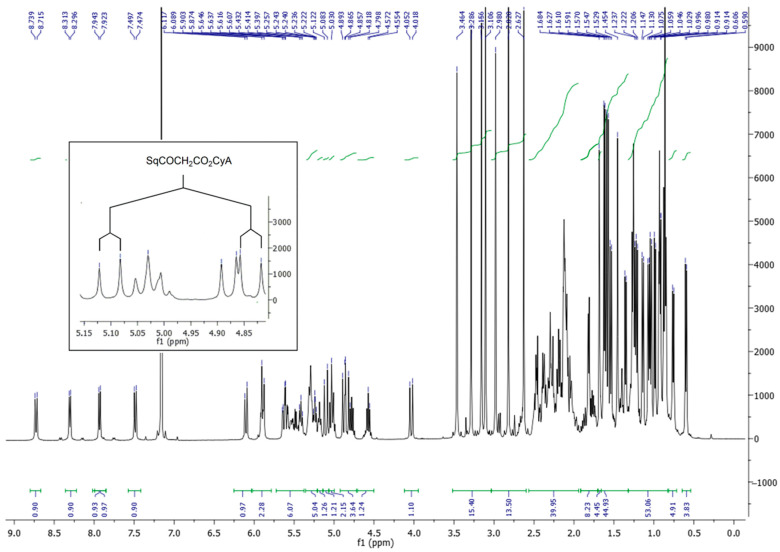
^1^H NMR of SqCsA (400 MHz, C_6_D_6_). The AB system of the methylene system; the glycolate linker is enlarged.

Extensive characterization was performed by IR spectrometry, ^1^H and ^13^C NMR including HSQC and HMBC 2D experiments and mass spectrometry. The SqCsA conjugate exhibited the characteristic absorption bands in the Fourier transform infrared (FTIR) spectrum for the C=O ester functions at 1769 and 1750 cm^−1^ as well as the strong absorption at 1620 cm^−1^ resulting from the amide bond of the CsA peptide core. The ^1^H spectrum in CDCl_3_ was found quite difficult to analyze due to the presence of conformers in slow interconversion [47]. A more straightforward spectrum was observed in C_6_D_6_ in which a major conformer (94%) was observed together with a minor component (6%) as evidence in the downfield part of the spectrum for the four NH signals (Figure 2). In addition to a few changes to the protons of cyclosporin A, the ^1^H NMR spectrum revealed the characteristic ethylenic protons of the five trisubstituted olefins of the squalene chain at 5.30–5.15 ppm together with the two multiplets at 2.51 and 2.32 assigned to the CH_2_CH_2_CO_2_ moiety. The H-C(β) of the MeBmt residue was shifted downfield at 6.05 ppm, as expected after acylation. The AB system of the glycolate linker appeared as two doublets with a 15.6 Hz coupling constant at 4.82 and 5.10 ppm, respectively. ^13^C NMR analysis revealed the presence of the two ester carbonyl groups at 168.2 and 172.4 ppm and the five double bonds of the squalene in addition to the two carbons of the double bond of the MeBmt amino acid in the range between 135 and 125 ppm. The covalent coupling with the squalenoyl moiety was further confirmed by ESI (+) mass spectrometry analysis, which showed the parent peak at *m*/*z* = 1665.2 corresponding to a monocharged [M+Na]^+^ ion together with the bicharged [M+Na]^2+^ ion at 844.1. High-resolution mass spectroscopy unambiguously confirmed the C_91_H_155_O_15_N_11_ formula of the bioconjugate.

### 3.2. Formulation and Characterization of SqCsA NPs

**Figure 3:**
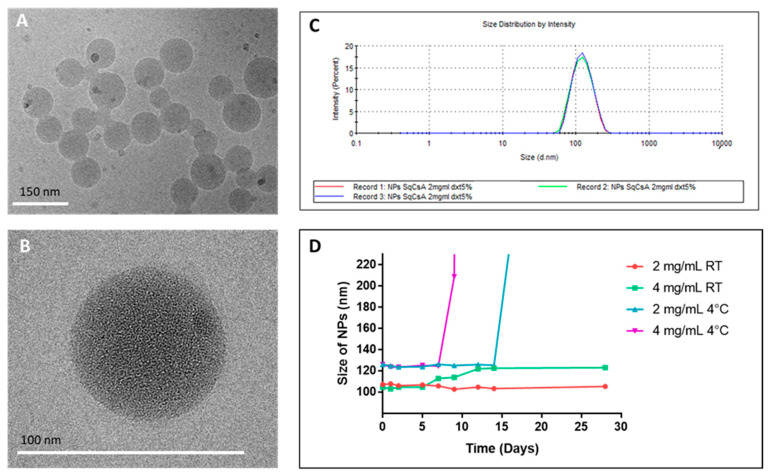
SqCsA nanoparticles (NPs) characterization by using cryogenic transmission electron microscopy (cryoTEM) (**A**,**B**) and dynamic light scattering (DLS) (**C**,**D**) of NPs suspensions. Stability assessment was performed for 28 days (**D**).

SqCsA NPs were obtained by nanoprecipitation of an ethanolic solution of a bioconjugate in a 5% dextrose aqueous solution. The covalent bond results in NPs with a high drug loading (ratio between molecular weights in percent) of 73%. CryoTEM images showed NPs with a spherical shape and size ranging from 60 to 150 nm with a mean diameter of approximately 105 nm (Figure 3A,B). These data were confirmed by dynamic light scattering (DLS) showing a unimodal size distribution with a mean diameter of 117 ± 1.11 nm and a polydispersity index of 0.09 (Figure 3C). The same size distribution can be found for multiple other Sq bioconjugates such as nucleoside analogues [32,38,48], anticancer drugs [33,35,49], or peptides [34].

Although other forms of lipid NPs of CsA exist (liposomes and solid lipid NPs), they lead either to larger NPs size/higher polydispersity index [22,50,51] or to a smaller size but with modest drug loading [52]. Furthermore, polymeric nanoparticles of CsA did not improve this general trend and exhibit identical characteristics as lipid NPs [53,54,55,56]. Our nanoparticles showed a surface zeta potential of −14.2 ± 0.65 mV, which remained stable over time, indicating an adequate colloidal stability. NP stability was assessed over 28 days at two concentrations (2 and 4 mg/mL) and under two storage conditions (4 °C and RT) (Figure 3D). The stability was evaluated based on visual inspection of the vials and evolution of the size measured by DLS. Storage at 4 °C was found not to be optimal because a precipitation occurred at day 9 for 4 mg/mL and day 21 for 2 mg/mL. By contrast, both NP concentrations stored at RT remained stable, with an increase in size between day 5 and 14 for 4 mg/mL. Our results revealed that SqCsA NPs possess interesting physical characteristics, since they combine a small size and narrow polydispersity, as well as a favorable colloidal stability at RT.

### 3.3. HPLC Analysis

**Figure 4:**
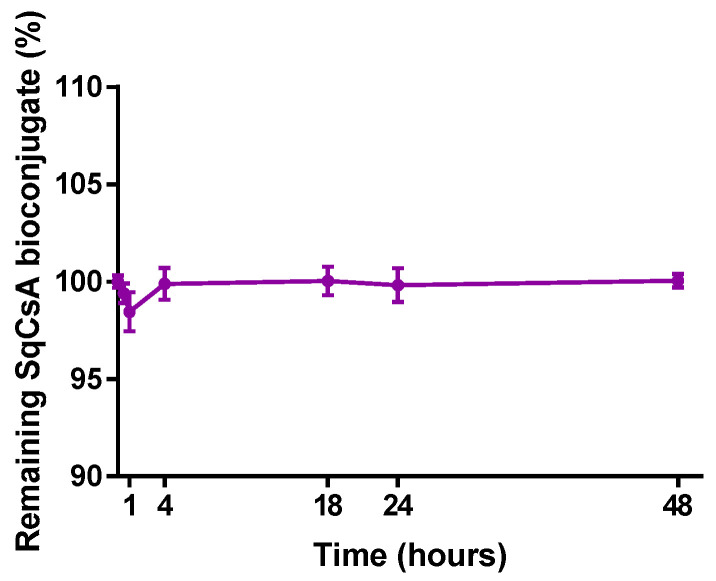
Remaining SqCsA bioconjugate in fetal bovine serum for 48 h. We see no degradation of the bioconjugate. Data represent mean ± SD of three replicates.

To evaluate the degradation of SqCsA bioconjugate and the release of CsA in serum, the amount of SqCsA bioconjugate, free CsA, and free squalenic acid were studied using HPLC. The drug release study was carried out in FBS incubated at 37 °C to adjust the method to more realistic conditions. The results (Figure 4) showed no significant decrease of SqCsA bioconjugates incubated in serum over 48 h. Moreover, we could not quantify any release of neither CsA nor squalenic acid from the SqCsA bioconjugates. These results are close to those obtained with the same type of NPs but with a different molecule such as adenosine/vitamin E [37]. However, our NPs appear even more stable than those with adenosine, which was most likely because CsA is more lipophilic. This observation has already been made for another type of lipid NP; in fact, the more lipophilic the molecule is, the slower the release will be [57]. Consequently, the NPs are stable, and the bond between CsA and squalenic acid is hardly hydrolyzable in FBS. Previously, our laboratory demonstrated that the same type of NPs but conjugated with antibiotics could lead to intracellular release of the drug [58]. This leads us to believe that if our NPs were internalized by the cells, CsA would be released into the cytoplasm within the cells.

### 3.4. Cytotoxicity of SqCsA NPs

**Figure 5:**
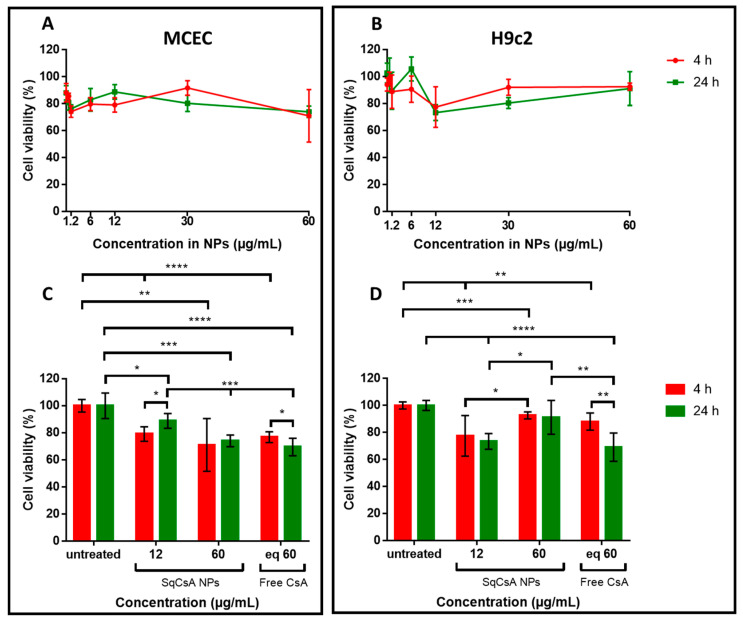
Cell viability assessment of Mouse Cardiac Endothelial Cells (MCEC) (**A**,**C**) and H9c2 (**B**,**D**) cell lines treated with SqCsA NPs. Cell viability is expressed as a percentage relative to the viability of untreated cells. Use of an equivalent (eq) concentration in free CsA (**C**,**D**). * *p* ≤ 0.05, ** *p* ≤ 0.01, *** *p* ≤ 0.001, **** *p* ≤ 0.0001.

Increasing concentrations of SqCsA NPs were tested on two different cardiac cell lines, H9c2 and MCEC, to determine their toxicity with an MTT assay. A free CsA solution at a dose equivalent to the highest concentration of NPs was used as a positive control. Cell viability was high at over 70%, regardless of NPs concentration and cell line (Figure 5A,B). Nevertheless, NPs were slightly cytotoxic but not more than free CsA (Figure 5C,D). Indeed, it has already been shown that free CsA can be cytotoxic at high concentrations on H9c2 cells [59]. Moreover, the effect is even more pronounced in MCEC cells (Figure 5C) as well as in another endothelial cell line [60].

### 3.5. Cell Uptake of Fluorescently Labeled SqCsA NPs

We evaluated the cellular uptake of NPs on two cardiac lines: H9c2 and MCEC. After incubation with two different concentrations of SqCsA NPs, the cells were fixed and analyzed at different times by using CLSM (Confocal Laser Scanning Microscopy).

**Figure 6:**
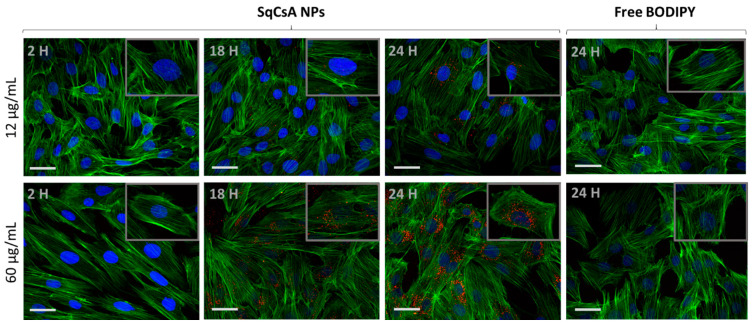
SqCsA NPs uptake assessment. The H9c2 cell line was incubated with two concentrations of nanoparticles 12 or 60 µg/mL loaded with Cholesteryl 4,4-difluoro-5-(4-methoxyphenyl)-4-bora-3a,4a-diaza-s-Indacene-3-undecanoate (CholEsteryl BODIPY™, red). After different times, cells were rinsed, fixed, and incubated with phalloidin (green). The cells were mounted with medium containing 4’,6-diamidino-2-phénylindole (DAPI, blue), analyzed under confocal microscope, and imaged. Cells incubated with only CholEsteryl BODIPY™, at an equivalent concentration to 60 µg/mL of NPs, were used as control and imaged at 24 h. Insert 90× magnification. Scale bar = 50 µm.

NP uptake by the two types of cardiac cells was cell type dependent but also depended on the incubation time. The uptake was observed as early as 120 min, which gradually increased with the incubation time. Our results showed a very low accumulation into H9c2 at 2 h but reached a plateau between 17 and 24 h when incubated with 60 µg/mL of SqCsA NPs (Figure 6).

**Figure 7:**
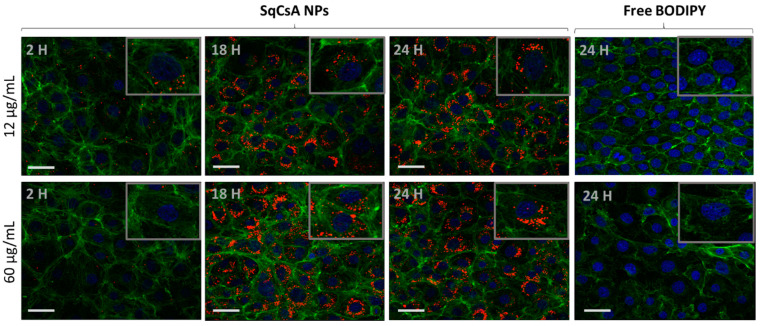
SqCsA NPs uptake assessment. The MCEC cell line was incubated with two concentrations of nanoparticles 12 or 60 µg/mL loaded with CholEsteryl BODIPY™ (red). After different times, cells were rinsed, fixed, and incubated with phalloidin (green). The cells were mounted with medium containing DAPI (blue), analyzed under confocal microscope, and imaged. Cells incubated with only CholEsteryl BODIPY™, at an equivalent concentration to 60 µg/mL of NPs, were used as control and imaged at 24 h. Insert 90× magnification. Scale bar = 50 µm.

In contrast, MCEC showed higher cellular uptake as early as 2 h (Figure 7). This could be explained by the different characteristics of these two types of cells. Endothelial cells possess a better capability for endocytosis [61,62,63], whereas this mechanism is not a characteristic of cardiomyocytes. Recently, Zhang et al. [56] found that the uptake of targeted poly(lactic-co-glycolic acid) (PLGA) -CsA NPs by H9c2 displayed time-dependency and was mainly achieved through macropinocytosis pathways. It would also be interesting to determine the mechanism through which SqCsA NPs enter these two cell types. Moreover, serial z-sections of the cells, each 1 µm thick, confirmed the detection of fluorescence in all the sections between 10 and 25 µm from the surface of the cells, indicating that the nanoparticles are effectively internalized by the cells and are not on the outer surface of the cell membrane.

### 3.6. Cardioprotective Effect Assessment

**Figure 8:**
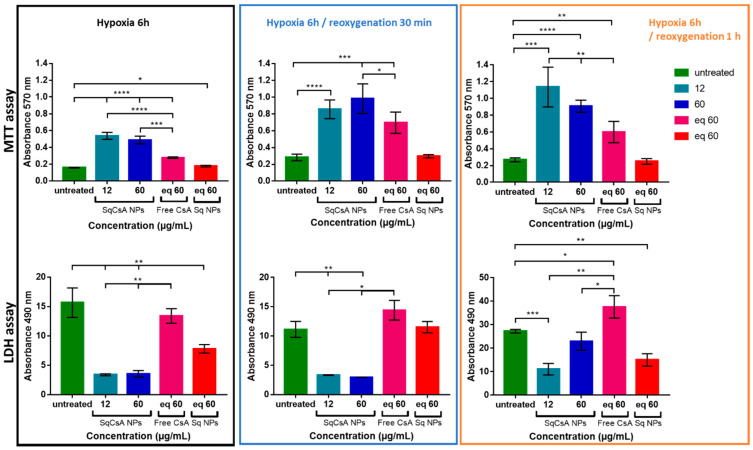
Cardioprotective effect assessment on H9c2 cell line. Cells were incubated with SqCsA NPs for 24 h. Controls (untreated cells, free CsA, or Sq NPs) were done under the same conditions. After incubation time, cells were rinsed with Phosphate-Buffered Saline (PBS) and incubated in hypoxic condition (1% O_2_ and restraint medium without fetal bovine serum (FBS) and glucose). Cardioprotection was assessed by using thiazolyl blue tetrazolium bromide (MTT) test (upper panel) and lactate dehydrogenase (LDH) test (lower panel). For the MTT tests, the higher the percentage, the more viable the cells. For the LDH tests, the higher the percentage of LDH-released protein, the more damaged the cells. * *p* ≤ 0.05, ** *p* ≤ 0.01, *** *p* ≤ 0.001, **** *p* ≤ 0.0001.

We assessed the ability of the new nanomedicines to protect cardiac cells from the deleterious effects of ischemia. mPTP is known to be a key mediator of cardiomyocyte death in the early phase of ischemia/reperfusion injury, as it remains closed during ischemia but opens rapidly in the first few minutes after cardiac reperfusion. The opening of mPTP induces potential mitochondrial membrane collapse, cytochrome C efflux, and finally cell death [64,65]. Based on the results obtained on cellular uptake under normoxic conditions, we incubated H9c2 and MCEC cardiac cell lines with SqCsA NPs 24 h prior to hypoxia. H9c2 cells were chosen for this study because they were found to be closer to normal primary cardiomyocytes in terms of energy metabolism characteristics and their sensitivity to hypoxia/reoxygenation [66]. Here, we analyzed cell viability at three time points: after 6 h of hypoxia, after 6 h of hypoxia followed by 30 min of reoxygenation, and after 6 h of hypoxia followed by 1 h of reoxygenation. The results obtained with the MTT test (Figure 8) showed a protective effect when H9c2 cells were incubated with SqCsA NPs at both concentrations (12 and 60 µg/mL), while no protective effect was observed on cells incubated with free CsA or only Sq NPs. This cardioprotective effect was even stronger when cells were reoxygenated for 30 min or for 1 h. Similar results were obtained with an LDH assay, which measures LDH release in culture supernatants upon cell lysis. Once again, our results highlighted a protective effect on cells incubated with SqCsA NPs compared to those untreated or incubated with free CsA or SqNPs (Figure 8). One explanation could be related to the fact that SqCsA NPs accumulate in the endosomes/lysosomes of the cells and that CsA is progressively released by the NPs and thereby could act at low drug concentrations in the mitochondria. Further experiments should be performed to quantify the accumulation of these NPs in the endosomes and to determine the mechanisms by which CsA is released from the NPs.

The strong inhibition of LDH release had previously been observed by Zhang et al. [56] when incubating CsA@PLGA-PEG-SS31 with H9c2 cells. It is noted that in this study, H9c2 cells were incubated with hypoxic culture medium for 3 h and treated with NPs 4 h before reoxygenation. In a study conducted on primary cardiomyocytes, cultures of 14-day old Sprague–Dawley rats were subjected to simulated ischemia for 4 h and reperfusion for 3 h. Under hypoxic conditions, the authors observed maximum cytoprotection after 3 h of reoxygenation at 50–100 nM. Increasing free CsA concentrations at 200 nM decreased cytoprotection [67]. The reason for this narrow therapeutic range is unclear, but it could be attributed to the non-specific inhibitory effects of CsA on cytosolic cyclophilin or the inhibitory effects of CsA on mitochondrial respiration [15]. Nazareth et al. [68] found protection on adult rat ventricular cardiomyocytes against necrotic cell death in a narrow therapeutic range (200 to 400 nM).

**Figure 9:**
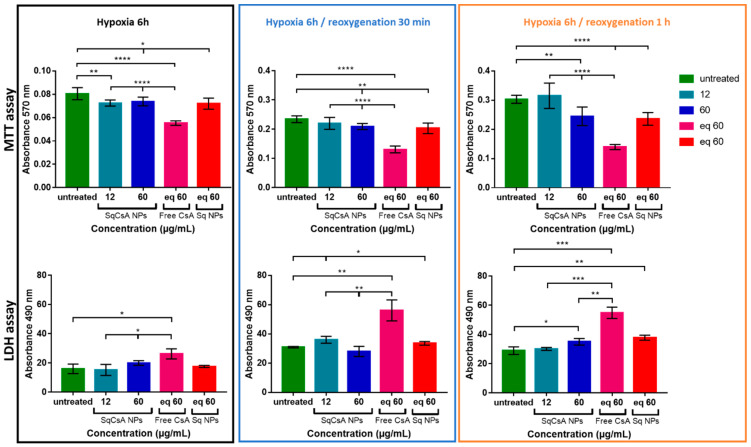
Cardioprotective effect assessment on MCEC cell line. Cells were incubated with SqCsA NPs for 24 h. Controls (untreated cells, free CsA, or Sq NPs) were done under the same conditions. After incubation time, cells were rinsed with PBS and incubated in hypoxic condition (1% O_2_ and restraint medium without FBS and glucose). Cardioprotection was assessed by using MTT test (upper panel) and LDH test (lower panel). For the MTT tests, the higher the percentage, the more viable the cells. For the LDH tests, the higher the percentage of LDH-released protein, the more damaged the cells. * *p* ≤ 0.05, ** *p* ≤ 0.01, *** *p* ≤ 0.001, **** *p* ≤ 0.0001.

The results obtained on MCEC were more mitigated (Figure 9). Even though SqCsA NPs showed almost no effect compared to untreated cells, they appeared to be less deleterious than free CsA. This could be explained by a greater absorption by these cells. Accumulation at high doses in endothelial cells would have a deleterious effect on the cells. Another explanation could be that a higher accumulation in the cell could lead to a higher release of cyclosporine and thus exceed the therapeutic window. Therefore, it would be interesting to test lower doses on this type of cells.

## 4. Conclusions

In conclusion, we obtained a new type of NPs based on the covalent bond between squalene and cyclosporin A. This bioconjugate formed NPs of controlled size in an aqueous medium. The NPs obtained were stable at room temperature for several weeks, and no significant release of CsA was found when incubated in FBS over 48 h. The cardioprotective capabilities of these NPs were evaluated on two cardiac cell lines subjected to experimental hypoxia/reoxygenation. They appeared to have a strong protective effect on cardiomyoblasts but a more mitigated one on endothelial cells, suggesting a possible hydrolysis of the ester bond between CsA and squalene inside the cells. Understanding the outcome of NPs in the cells and the mechanisms of cyclosporin release from NPs is essential for a possible future medical application.

## Data Availability

Not applicable.
